# Feasibility and perceptions of a benzodiazepine deprescribing quality improvement initiative for primary care providers in Japan

**DOI:** 10.1186/s12875-024-02270-2

**Published:** 2024-01-24

**Authors:** Masahiro Nishimura, Alan R. Teo, Takahiro Mochizuki, Naoki Fujiwara, Masakazu Nakamura, Daisuke Yamashita

**Affiliations:** 1 Japan Association for Development of Community Medicine (JADECOM), 15th floor, 2-6-3 Hirakawa-cho, Chiyoda-ku, Tokyo, 102-0093 Japan; 2 Oregon Health and Science University (OHSU), Portland, United States; 3 VA Portland Health Care System, Portland, United States

**Keywords:** Quality improvement, Benzodiazepines, Primary health care, Japan

## Abstract

**Background:**

Quality improvement (QI) initiatives in primary care in Japan are rare. One crucial area for QI is the appropriate prescription of benzodiazepines due to the large and growing elderly population in the country.

**Objective:**

This study aimed to determine the feasibility and other perceptions of a Benzodiazepine receptor agonist medications (BZRAs) deprescribing QI initiative for primary care providers (PCPs) in Japanese primary care clinics.

**Design:**

A qualitative study within a QI initiative.

**Participants:**

We recruited 11 semi-public clinics and 13 providers in Japan to participate in a BZRAs deprescribing initiative from 2020 to 2021. After stratifying the clinics according to size, we randomly allocated implementation clinics to either an Audit only or an Audit plus Coaching group.

**Interventions:**

For the Audit, we presented clinics with two BZRAs-related indicators. We provided monthly web-based meetings for the Coaching to support their QI activities.

**Approach:**

After the nine-month initiative, we conducted semi-structured interviews and used content analysis to identify themes. We organized the themes and assessed the key factors of implementation using the Consolidated Framework for Implementation Research (CFIR) framework.

**Key results:**

Audit plus Coaching was perceived as more valuable than Audit only intervention. Participants expressed intellectual curiosity about the QI initiative from resources outside their clinic. However, adopting a team-based QI approach in a small clinic was perceived as challenging, and selecting the indicators was important for meaningful QI.

**Conclusion:**

The small size of the clinic could be a potential barrier, but enhancing academic curiosity may facilitate QI initiatives in primary care in Japan. Further implementation trials are needed to evaluate the possibility of QI with more various indicators and a more extended period of time.

**Supplementary Information:**

The online version contains supplementary material available at 10.1186/s12875-024-02270-2.

## Background

For decades, Japan has maintained its status as a country with one of the highest life expectancies [1]. Japan also ranked 11th among 195 countries in healthcare access and quality [2]. However, long-term sluggish economic growth and an aging population are forcing Japan to increase productivity in all sectors [1]. The OECD Review of Health Care Quality 2014 pointed out two challenges in monitoring and improving health care quality in Japan: the first is that few quality initiatives are organized at a system level, the second is that although there are many quality-related activities at an individual level, these are haphazardly applied [3]. A striking feature of the Japanese health system is its openness and flexibility [3]. However, in an aging society with limited national financial affordability, current light-touch governance without surveillance is not sustainable. Implementing QI embedded in daily practice is needed to shift the Japanese Primary Care system from a traditional volume-based system to a quality-based. The Japan Council for Quality Health Care launched the “System Improvement Project for Quality Improvement of Medical Care [4]” in 2019, supported by the Ministry of Health, Labor and Welfare. However, the primary target of the government-led project is mid- to large- size hospitals, and QI in Primary Care has not been well discussed.

Several structural barriers impede the implementation of QI activities at a system level in Japanese Primary Care. Firstly, the payment system operates on a fee-for-service basis. Secondly, most clinics are solo practices owned by physicians or small medical corporations, with limited transparency in practice outcomes [5]. Thirdly, the Primary Care training system was not formally established until 2018, when the Japanese Medical Specialty Board approved “General Medicine” as the 19th essential medical specialty [6]. Most of Primary Care is provided by former specialists who transitioned to general practice, and the practice patterns and scopes widely vary [7–8]. Lastly, the electronic health care records in Primary Care have disseminated without the requirement of interchangeability of clinical data or the third-party evaluation [9]. Consequently, the environment of QI activities at a system level has not been fostered.

Appropriate usage of benzodiazepine sedatives and hypnotics is essential, especially for those living in an aging country like Japan. Benzodiazepine receptor agonist medication (BZRAs) increases the risk of falls and cognitive impairment among older patients [10]. According to the International Narcotics Control Board, Japan is the 10th largest country in BZRAs consumption among all 35 OECD countries [11]. If we focus on only benzodiazepine-type hypnotics, Japan is second. Moreover, etizolam, clotiazepam, and z-drugs (zolpidem, zopiclone, eszopiclone, and zaleplon), which consist of 42.6% share of total BZRAs annual prescriptions in Japan [12], are not included in these statistics. Therefore, the number of BZRAs prescriptions may be underreported. The Ministry of Health, Labor, and Welfare commenced the policy with negative incentive of reimbursement against long-term (over 12 months) prescriptions of psychotropic drugs, including BZRAs, in 2018 [13]. However, the pre-post study reported no significant change (Apr 2014-Mar 2015: 10.7%, Apr 2018-Mar 2019: 10.7%) [14]. In 2021, the OECD HCQO (Health Care Quality Outcome) included “Elderly patients with prescription of long-term benzodiazepines or related drugs” as one of the 64 QI indicators, which should be measured as universal indicators [15]. However, Japan has not submitted this data.

The QI project for deprescribing benzodiazepine in the US indicated that combined pharmaceutical and academic detailing was effective [16–17]. A controlled trial conducted in Australia showed a sustained effect on the reduction in the use of benzodiazepine in a 6-month intervention (including medication audit and feedback, educational sessions for staff and interdisciplinary sedative review) [18]. We found no prior study investigating the QI activities related to benzodiazepine deprescribing in Japanese Primary Care setting nor qualitative research related to QI projects for deprescribing benzodiazepines.

The study aims to determine the feasibility and other perceptions of QI initiative for PCPs in Japan using a BZRAs deprescribing as a topic of QI.

## Methods

### Setting and sample

The study was conducted under the JADECOM (Japan Association for Development of Community Medicine) research institute. JADECOM runs 40 clinics in rural areas where medical resources are scarce [19]. The local government partly funds clinics to provide medicine in the community. JADECOM established PBRN (Practice-Based Research Network) in 2018. We recruited study clinics and providers through the monthly meetings and mailing list and enrolled 11 participating clinics and 13 providers. We designated two pilot clinics and piloted the whole intervention process for the first three months (April 2020-June 2020). Then, we stratified nine clinics according to the number of providers (five solo clinics or four more than two providers’ clinics) and randomized each group to Audit only clinics or Audit plus Coaching clinics. Randomization has been conducted by using an online web service [20]. As a result, we had four Audit only clinics and five Audit plus Coaching clinics (Table 1).

**Table 1:** Characteristics of Participating Clinics and Providers

Clinic	Average numbers of outpatient per day	Numbers of providers at each clinic	Experience of providers (PGY)	Intervention group	Interviewed
A	43	1	8	Audit only	Yes
B	13	1	36	Audit plus coaching	Yes
C	20	1	19	Audit plus coaching	Yes
D	15	1	35	Audit plus coaching	Yes
E	32	1	36	Audit only	Yes
F	36	2	10	Audit only	Yes
G	80	2	13	Audit only	Yes
H	16	2	19	Pilot	No
			8		No
I*	19	2	13	Audit plus coaching	No
J	22	1	35	Pilot	No
K	108	3	37	Audit plus coaching	Yes
			23		Yes
Median	22	1	19		

PGY: Post Graduate Year

* dropped out

### Implementation

At the beginning of the intervention, we offered 2-hour didactic lecture about the evidence-based practice of insomnia and anxiety-related disease and the appropriate use of BZRAs to align the knowledge of participants. Secondly, we established the workflow in every clinic that transferred their Health Insurance Claims Data, including the BZRAs’ prescription data, to the PBRN data center every month. Aggregate information was de-identified, and investigators and participants could not access a patient ID. Thirdly, the data center extracted the indicators as below. The list of BZRAs we defined in the research is shown in Supplementary 1.

#### Quality indicator 1

The percentage of patients prescribed BZRAs (number of patients who are prescribed BZRAs per month / number of patients who are prescribed any medication per month).

#### Quality indicator 2

The average prescribed tablets of BZRAs per patient panel (number of all tablets of BZRAs in the clinic per month / number of patients who are prescribed any medication per month).

**Fig. 1:**
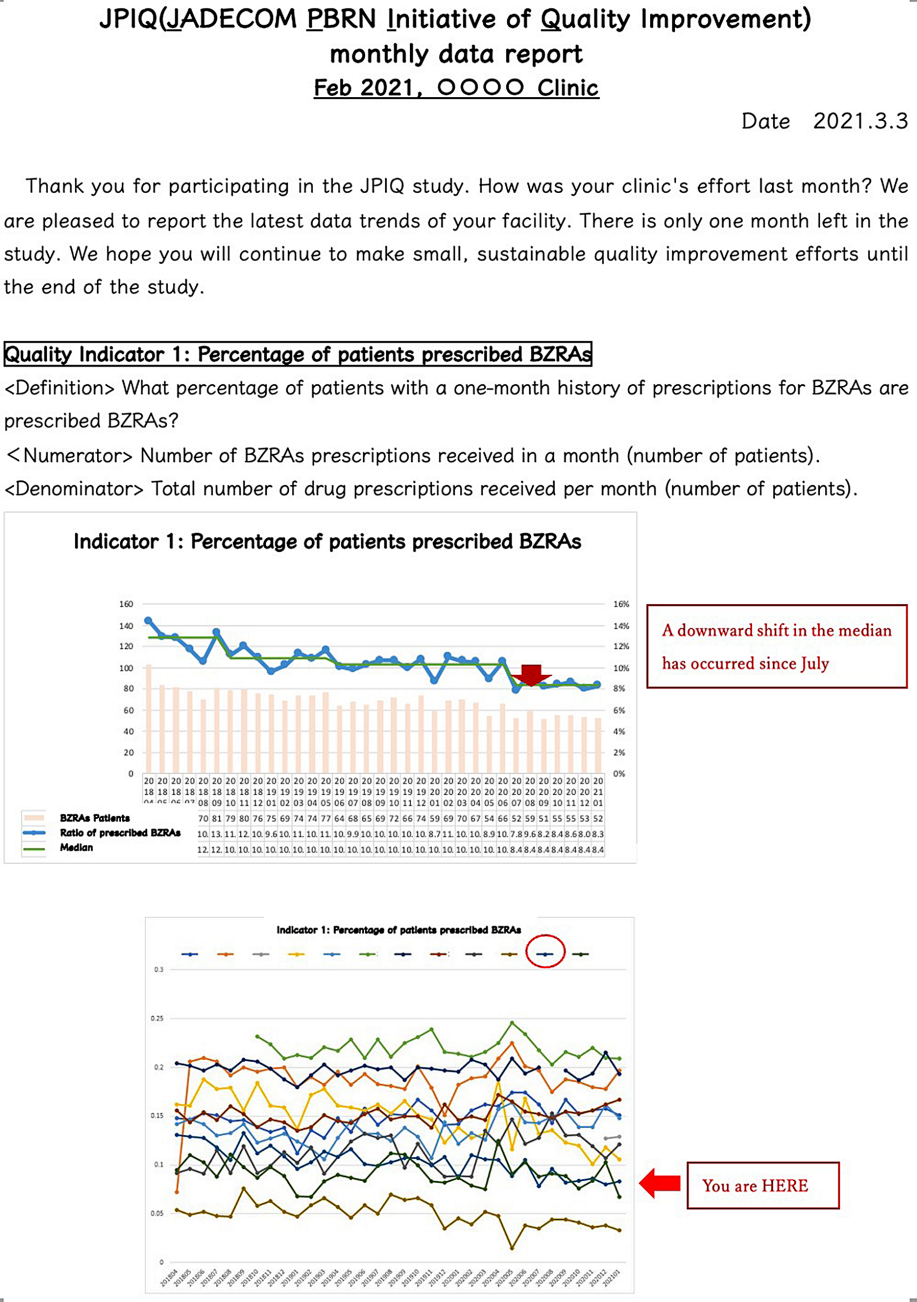
Sample of monthly data report

Fourthly, the lead author created run charts of quality indicators 1 and 2, respectively, and sent them back to every clinic electronically titled “monthly data report” (Fig. 1) (**Audit**). The lead author also provided 60 min of web-based meetings (**Coaching**) to the Audit plus Coaching group monthly. The lead author offered knowledge-based lectures based on IHI (Institute of Health Improvement) [21] e-learning materials in the web meeting and coached their QI process. We implemented the intervention for nine months (July 2020 to March 2021). One clinic allocated as an Audit plus Coaching group has dropped out due to an increased workload related to the COVID-19 pandemic.

### Evaluation and analysis

After the implementation, we asked all nine providers of the intervention group who had agreed to participate in the research at the beginning of the initiative to take the semi-structured interview (May to July 2021). The interview was conducted to get their perception of the QI experience and thoughts about the initiatives. The lead author (MNi) interviewed 4 participants in the Audit only group. The two co-authors, TM and DY, interviewed 5 participants of the Audit plus Coaching group. All three interviewers were members of the JADECOM-PBRN, male family physicians, and had enough knowledge and experience in Primary Care in Japan. DY worked as a family physician in the US as well. They used the original interview guide (Supplementary 2) we developed for the study. Each interviewer held a 26–51 (avg. 39) minute online interview by Microsoft Teams®, recorded the conversation, and transcribed it into text using software (Notta.©) with manual correction. We conducted deductive and inductive content analysis to identify themes grounded in a priori categories and unexpected themes [22]. We first utilized a deductive analytic approach, creating a preliminary codebook organized by interview guide content, with MNi and DY independently double-coding each interview. Then, led by a researcher with extensive qualitative experience (AT), the analytic team (MNi, TM, and DY) used an inductive approach based on an iterative review of codes, analytic memos, and team-based discussions. We used NVivo (Ver 1.7.1)© software for the analytic process. Next, we used the Consolidated Framework for Implementation Research (CFIR) [23] as an analytic framework because we considered CFIR, which is a pragmatic meta-theoretical framework that can be used to complement these theories with its comprehensive taxonomy of specific constructs related to the intervention, inner and outer setting, individuals, and implementation process [23], was helpful in organizing the results of the study. We examined the output from codes and organized data into five domains of CFIR. Using this analytic framework, we identified and synthesized themes within those categories and selected quotations that exemplified themes.

### Ethical consideration

All methods were carried out in accordance with the declaration of Helsinki, and Ethical Guidelines for Medical and Biological Research Involving Human Subjects by the Japanese government. Informed consent was obtained from the patients by opt-out documentation. All study protocols were approved by the IRB of JADECOM (20200312-03) and OHSU (STUDY00023408).

## Results

### Participants

The characteristics of the interviewees are shown in Table 1. All participants were Japanese males and worked as PCPs in the community. The participants’ educational backgrounds varied, from young providers who graduated from the family medicine residency program to senior providers who have trained independently without the residency system. The median length of a career as a physician was 19 years (range:8–37). The size of participated clinics was small: the median number of providers per clinic was 1, and the median number of outpatients per day was 22. The interviewees and participants had known each other online prior to the survey, but had never worked together in a clinical setting, except for MNi and one participant. About half of the participants knew the term “QI in medicine,” but others did not. All of them, including those who did not know the concept of QI, expressed their prior experiences in improving clinical work. Still, none of them had had the experience of taking the education or intervention of QI before. Table 2 provides themes that emerged through the interview’s content analysis organized by the CFIR framework.

**Table 2:** Themes from Contents Analysis

CFIR domain	CFIR subdomain S	Theme	Comment
Intervention characteristics	Design quality & packaging	The Audit was feasible but insufficient because the number of indicators without any interpretation or discussion was less meaningful for practitioner	*“I thought it was difficult to decide whether the data of my clinic was the reasonable or not. Each clinic must have their own background and reason, so I think it is nonsense to compare the number without any discussion.” (a)*
		During a busy practice, paying attention to the QI report felt like a bother and interruption of their limited time	*“I could not recognize the meaning of the indicators at a glance. I have to admit that I gave up taking time and trying to understand the meaning at such a moment.” (f)*
	Evidence quality	Selecting the theme and indicators was another challenging issue for meaningful QI	*“I noticed that there were patients who we can never change their belief. They have their own story and reason about taking BZRAs in their life, and I think it is not good practice to persuade them to stop the medication at the cost of the patient-doctor relationship.” (c)*
Outer setting	External policy & incentives	Most participants in both group appreciated and favored the organizational QI support from outside resources	*“If the government has the strict rule to survey BZRAs metric, we may try to improve desperately.” (e)*
			*“Sometimes it is difficult to begin something new in a small clinic because we have hierarchical relationships; I mean, staff might feel QI as irresistible pressure from the leader. I think outer group people like you are more neutral to initiate this type of work.” (k2)*
Inner setting	Structural Characteristics	Characteristics of QI in Primary Care in Japan tend to be physician-centered	*“You know, we cannot act as a team in such a small clinic. Generally, our practice heavily relies on the physician’s knowledge and attitudes.” (d)*
		Many participants said it was challenging to find time to increase their effort to improve.	*“QI would be difficult if we don’t have enough time in busy schedule, or our own good health condition.” (d)*
	Implementation climate	Providers have discretion and flexibility to adjust participation levels on a flexible basis	*“Thankfully, we, PCPs have much autonomy about our work. When we are busy, we save our energy to do more urgent care. And if we have time to do the improvement work, we are happy to share more time for the better practice.” (e)*
		All participants mentioned that submitting the claims data regularly was feasible	*I was a little confused at first, but once I got to the first part, all I had to do was send it to the office and they sent it to me, so I guess there were no more hurdles there. (e)*
Characteristics of individuals	Knowledge & beliefs about the intervention	Participants tended to evaluate themselves according to their performance in the past or predecessors, but not their performance during the intervention	*“The practice of the predecessor was not so good, so there was room to improve in my clinic.” (f).*
			*“I have been doing the improvement work since before this project, so there was no room for me to improve anymore.” (a, c)*
	Other personal attributes	Providers valued QI activities as a chance to review their practices objectively. They also showed an intellectual curiosity for continuous self-learning and the opportunity to participate in scholarly activities	*“It was great to see my clinical performance objectively in that way. It is easy for us to fall into static status because solo practitioners like me always work in a closed room without judgment from others. I sincerely thought it was important to learn and discuss with others about my practice.” (d)*
			*“In my time, I avoided or turned away from such clear and explicit performance in the practice, so I was strongly impressed by this academic approach.“(b)*
Process	External change agent	Participants of Audit plus Coaching group concerned that Coaching was highly dependent on the personal skills of the coach	*“We need a good coach. Otherwise, we might loosen our interest in participating the activities.” (k1)*

CFIR: Consolidated Framework for Implementation Research

QI: Quality Improvement

### Overview of qualitative findings

Most participants showed a positive attitude and curiosity towards the concept of QI in terms of its novelty and academic aspects. Interestingly, the senior providers expressed more positive comments than the younger providers. Additionally, the Audit plus Coaching group participants were likely to say the intervention was a meaningful activity for PCPs, although the Audit only group had a negative impression of the intervention. They also expressed that the regular submission of claims data was feasible, and the QI intervention was pragmatic only if a trustable and sustainable outer team supported them.

As for effectiveness, we found that the Audit and/or Coaching influence on their clinical practice was limited. They said it was challenging to find the time and pay attention to the additional work in their daily practice. We also discovered that the concept of QI, which is based on a team-based approach and system thinking, was challenging in the small clinic. Another barrier was the difficulty in selecting the theme and indicator. Every provider acknowledged that deprescribing inappropriate BZRAs is essential but felt it is hard for them to control the prescription pattern because the inappropriate usage of BZRAs tends to be related to issues such as physicians-patient relationship, individual thought and the norm of the society. They added that most patients have strong beliefs about taking pills and hesitate to decrease or stop the medication, and deprescribing BZRAs does not always equal the best practice for everyone.

#### CFIR framework 1: intervention characteristics

Participants of both groups said the Audit was feasible but insufficient because the number of indicators without any interpretation or discussion was less meaningful for practitioners. Participants of the Audit only group mentioned they had difficulty evaluating and interpretating the data in their practice. One participant was concerned that showing a bad outcome motivates PCPs negatively. On the other hand, participants of the Audit and Coaching group said the discussion in the Coaching session was valuable and thankful, adding the meaning of the data in the QI report.

Another issue related to the design was the appearance of the QI report. One of the reasons why they utilized the Audit infrequently was that the participants perceived the graph’s outlook and the definition of indicators as complicated. They expressed that during a busy practice, paying attention to the QI report felt like a bother and interruption of their limited time.

Selecting the theme and indicators was another challenging issue for meaningful QI. Participants said that changing the prescription behavior of BZRAs was a fundamentally difficult topic for any clinician because BZRAs were the medication for behavioral health and mental conditions such as insomnia and anxiety. Some patients were under the condition of addictive status, so it was a complex task for them to improve.

#### CFIR framework 2: outer setting

Most participants in both groups appreciated and favored the organizational QI support from outside resources because it saves providers extra time and effort to improve practice. One participant said initiative from outside was helpful because it prevented hierarchical overpressure on staff from a physician in a small clinic. Other participants mentioned that external incentives like positive or negative financial payment systems or public surveillance from the government might facilitate providers’ motivation to improve their performance.

#### CFIR framework 3: inner setting

Although the lead author explained and facilitated the team-based approach to the Coaching group, many participants said most of their activities were conducted within PCPs’ actions or attitudes in their practice, and system-level approaches were rare. In the Audit only group, we found no team-based approaches for improvement. When we compared solo clinics or clinics with multiple providers, PCPs in solo clinics were likelier to say they had the sense to handle the QI activity. PCPs in clinics with multiple providers said asking the other providers for more proactive participation was challenging. They are accustomed to making decisions individually, which means the characteristics of QI in Primary Care in Japan tend to be physician-centered.

Issues related to time and workload are classic but crucial for QI [24]. Many participants said it was challenging to find time to increase their efforts to improve. At the same time, some providers mentioned positive comments in terms of clinicians’ involvement between the constraints of time and effort. In addition, all participants mentioned that submitting the claims data regularly was feasible because the submitting process was straightforward, and administrative staff could do it independently.

#### CFIR framework 4: characteristics of individuals

The characteristics of the individual played a critical role in the improvement. We found that most participants tended to evaluate themselves according to their performance in the past or predecessors, but not their performance during the intervention.

The participants who achieved a better outcome than others mentioned,

*The predecessor’s practice was not so good, so there was room to improve in my clinic.*

However, the participants who achieved the worst outcome remarked,

*I have been doing the improvement work since before this project, so there was no room for me to improve anymore.*

Also, we found several positive personal attitudes about QI. Providers valued QI activities as a chance to review their practices objectively. They also showed an intellectual curiosity for continuous self-learning and the opportunity to participate in scholarly activities. Participants of the Audit plus Coaching group were more likely to appreciate that Coaching was valuable and novel because it is a rare opportunity for PCPs in small clinics to discuss the practice with others.

#### CFIR framework 5: process

Participants of the Audit plus Coaching group were concerned that Coaching was highly dependent on the personal skills of the coach. They mentioned that the flexibility of meeting times and the coach’s characteristics have influenced the participant’s motivation and QI performance. They are concerned with the generalizability and sustainability of the Coaching.

## Discussion

This is the first challenge to assess the feasibility and perception of the QI initiative in Japanese Primary Care related to BZRAs’ deprescribing. The study was conducted in a small clinic, and a wide range of participants’ experiences reflected in years of practice, representing the typical Primary Care setting in Japan. Through the study, we implemented the anonymous claim data-gathering workflow, which could be the foundation of other QI initiatives in this area.

Participants in both groups expressed their experience that only Audit was feasible, but insufficient to change their practice. And additional Coaching for interpretation and discussion was favorable for the meaningful QI. Systematic reviews indicated that Audit and feedback were more effective when provided both verbally and in written format [25]. Additionally, practice facilitation can significantly influence the adoption of evidence-based guideline in Primary Care.This effect is particularly strong when interventions are tailored and implemented with high intensity [26]. It sounds natural that providers were interested in a more customized approach than a data-driven approach, but it is important to know that they still favor learning even though Coaching is a more time-consuming intervention. The awareness of balancing standardization and customization is one of the takeaways from the experienced QI countries [27]. We observed a similar perspective in this initiative. On the other hand, the PCPs in the Audit plus Coaching group are more likely to be concerned about the feasibility of the intervention. Whether the external QI team is sustainable is the critical factor for the QI at a system level in Primary Care.

The selection of indicators was another issue that impacted the motivation of participants. Generally, each country’s public or academic groups create the appropriate clinical indicators, such as the National Quality Forum (NQF) [28] in the US and HCQO [15] in the OECD organization. Currently, no practical quality indicators encompass BZRA prescriptions in Japan. Significant and clinically relevant indicators of the appropriate BZRAs might incorporate patient safety and patient experience. However, considering the feasibility, we needed to choose the prescription pattern readily available from regular billing data. A national consensus for defining quality indicators in Primary Care is needed to enhance QI activity.

All participants agreed that the initiative was feasible daily in the practice if an outside resource guided and supported them. Matsumura et al., who developed the QIPC-J(Quality Indicators for Primary Care Practice in Japan) [29] which consists of 39 comprehensive indicators in a sophisticated way, reported that implementing QIPC-J in real-world clinical settings was highly time-consuming, primarily when they conducted a medical chart review [29]. We overcame this issue by avoiding medical chart review as the data-gathering method. However, the feasibility of Coaching is still a challenging issue.

The size of the clinic could be another barrier to team-based QI in Primary Care. In small clinics, the clinic’s practice is closely tied to the practice of personal PCPs. Generally, small clinics tend to have a conservative, paternalistic, hierarchical culture promoting physician-centered practice [30]. Therefore, there is little room for other professions to comment, including prescribing patterns and behavioral change. Considering that most clinics in Japan are solo-practice, engaging in QI at a system level could be a significant challenge. However, small-size clinics do not necessarily demonstrate a poor outcome. The Evidence NOW Initiative to promote evidence-based cardiovascular disease in Primary Care found that small-size and clinician-owned practices contributed to better blood pressure outcomes and tobacco cessation in primary care clinics [31]. It is suggested that if the clinician considers and tailors operational expectations to the practice setting, they can rapidly reach meaningful improvement. The disadvantage of a structural characteristic of Japan could become a strength if we can effectively encourage providers to change their practice. This provides a unique opportunity in Japan.

Curiosity and a positive attitude toward the academic aspects of QI activity may be the potential strength of Japanese PCPs. Systematic review and meta-analysis of practice facilitation within Primary Care settings indicated that alignment with professional values and intrinsic motivation was one of the positive perceptions of the value of performance measurement [32]. Ironically, the lack of academic focus and the delay of national-level quality control policy in Japan may make the PCPs think of this activity more positively. There are several negative perspectives from those countries that have already adopted the value-based-payment system, such as the US or the UK, that financial incentives have a small impact on care delivery [33]. Currently, Japan does not rely on financial incentives, which may help the PCP’s perception become positive and encourage a professional attitude toward QI. Approaching the QI interventions as opportunities to learn new knowledge and skills may facilitate further adaption.

There are several limitations to the interpretation of this study. First, all participants were acquaintances of researchers who interviewed them and members of the same PBRN group. Participants might have stated more polite or complimentary comments to investigators. Likewise, the study participants were all hired by the same organization. Considering the typical style of primary care clinics in Japan is a physician-owned clinic, we need special attention to adapt the outcome to other settings. Thirdly, the period of intervention was only nine months. It might be too short for PCPs to take action and get a sense of meaningful change in their practice. Lastly, this implementation period started in April 2020, so it is estimated that the pandemic of COVID-19 could have distracted their attention and time.

## Conclusion

The small size of the clinic and the feasibility of QI support from outside could be a potential barrier. Enhancing curiosity toward the academic aspect of QI and thoughtful intervention design may facilitate the implementation of QI initiatives in Primary Care in Japan. Further implementation trials are needed to evaluate the possibility of QI with various indicators and a more extended period.

### Electronic supplementary material

Below is the link to the electronic supplementary material.

**Supplementary Material 1:** Definition of BZRAs (Benzodiazepine receptor agonist medications)

**Supplementary Material 2:** Interview Guide

## Data Availability

Not applicable.

## Abbreviations

BZRAs: Benzodiazepine receptor agonist medications
CFIR: Consolidated Framework for Implementation Research
COVID-19: Coronavirus disease 2019
HCQO: Health Care Quality Outcome
IHI: Institute of Health Improvement
JADECOM: Japan Association for Development of Community Medicine
NQF: National Quality Forum
OECD: Organization for Economic Co-operation and Development
PBRN: Practice-Based Research Network
PCPs: Primary Care Providers
QI: Quality improvement
QIPC-J: Quality Indicators for Primary Care Practice in Japan
