# Supplementary material for: Feasibility and perceptions of a benzodiazepine deprescribing quality improvement initiative for primary care providers in Japan
Source: BMC Prim Care. 2024 Jan 24;25:35. doi: 10.1186/s12875-024-02270-2 (PMC10807085; doi:10.1186/s12875-024-02270-2)
Supplement: Supplementary file 1 — Supplementary Material 1: Definition of BZRAs (Benzodiazepine receptor agonist medications) [file 12875_2024_2270_MOESM1_ESM.docx]

**Supplementary 1**

**List of BZRAs(Benzodiazepine receptor agonist medications)**

| **Medication** | **YJ Code*** |
| --- | --- |
| Zopiclone | 1129007 |
| Zolpidem | 1129009 |
| Eszopiclone | 1129010 |
| Rilmazafone | 1129006 |
| Tandospirone | 1129008 |
| Estazolam | 1124001 |
| Flurazepam | 1124002 |
| Nitrazepam | 1124003 |
| haloxazolam | 1124005 |
| Triazolam | 1124007 |
| Flunitrazepam | 1124008 |
| Brotizolam | 1124009 |
| Lormetazepam | 1124010 |
| Oxazolam | 1124013 |
| Cloxazolam | 1124014 |
| Clorazepate Dipotassium | 1124015 |
| Diazepam | 1124017 |
| Fludiazepam | 1124019 |
| Bromazepam | 1124020 |
| Medazepam | 1124021 |
| Lorazepam | 1124022 |
| Alprazolam | 1124023 |
| Flutazolam | 1124024 |
| Mexazolam | 1124025 |
| flutoprazepam | 1124027 |
| Chlordiazepoxide | 1124028 |
| Ethyl loflazepate | 1124029 |
| Quazepam | 1124030 |
| Flunitrazepam | 1124400 |
| Midazolam | 1124401 |
| Diazepam | 1124402 |
| Bromazepam | 1124700 |
| Diazepam | 1124701 |
| Clonazepam | 1139003 |
| Clobazam | 1139006 |
| Etizolam | 1179025 |
| Clotiazepam | 1124026 |

*YJ Code^1)^ : Individual Drug Codes in Japan

1) KIMURA Eizen, UENO Satoshi, Trends in health information and communication standards in Japan. J. Natl. Inst. Public Health, 69 (1) : 2020 Available at https://www.niph.go.jp/journal/data/69-1/202069010007.pdf
