# Supplementary material for: Feasibility and perceptions of a benzodiazepine deprescribing quality improvement initiative for primary care providers in Japan
Source: BMC Prim Care. 2024 Jan 24;25:35. doi: 10.1186/s12875-024-02270-2 (PMC10807085; doi:10.1186/s12875-024-02270-2)
Supplement: Supplementary file 2 — Supplementary Material 2: Interview Guide [file 12875_2024_2270_MOESM2_ESM.docx]

**Supplementary 2**

**Interview guide**

Interview Setting

**Interviewee**

8 facilities and 9 participating physicians.

| Interviewee | Facility | Group | Interviewer |
| --- | --- | --- | --- |
| A | A | Audit only | MNi |
| B | B | Audit +Coaching | TM |
| C | C | Audit +Coaching | TM |
| D | D | Audit +Coaching | TM |
| E | E | Audit only | MNi |
| F | F | Audit only | MNi |
| G | G | Audit only | MNi |
| H | H | Audit +Coaching | TM |
| I |  |  | DY |

**Interviewer**

MNi(4)，TM(4)，DY(1)

**Interview Plan**

Date : 5/3/2021 ~ 7/9/2021

Device：Online video interview by Microsoft Teams

Interview Time： 30-60 min

Recording：

“Recording on Teams” Plus “Voice recorder”

Dictation：

”Notta”(i phone app.) Plus manual correction

Structured Interview Questions **For Clinical Audit Only Group**

| Category | Sub category | Question |
| --- | --- | --- |
| Opening | Ice breaking | Thank you for the participation of the interview. Do you have enough time and space? Please relax... |
|  | Recording + Consent | Obtain consent |
|  | Original experience and thoughts on appropriate usage of BZRAs and QI | Before this intervention begins, tell us your thoughts and experiences regarding the appropriate usage of BZRAs and Quality Improvement in medicine? |
| Clinical Audit | Experience(of Clinical Audit) | Please recall your monthly QI report. What intervention did you actually receive? What did you think or feel about the intervention at the time? (Please display the monthly QI report for each facility on the screen.) |
|  | Probe | How did you feel about receiving the data?  What did you think of the way the reports were sent?  How much did you actually look at the report?  What did you think when you saw the contents (graphs/reports)?  How easy to read?  What did you think about the appropriateness of the indicators? |
|  | Experience(of QI) | What did you actually do as QI activities during the intervention? |
|  | Probe | Personally? As a team? Have there been any changes to the system? |
|  | Feasibility | Feasibility (Is it realistically possible to continue Clinical Audit as a part of our daily work in the future? (In other words, can you participate in it without difficulty?) What do you think about the feasibility of Clinical Audit? |
|  | Enabler / Effectiveness | What do you think of the benefits and effectiveness of Clinical Audit? |
|  | Barrier / Problem | What do you think about barriers? workload? mentality? |
| Closing | Open question | Is there anything you would like to add at the end? Please feel free to share anything you would like to state based on this experience. |
|  | Probe | What are your thoughts on implementing (systematic and organized) QI activities in primary care in Japan? What do you think is needed to improve the quality in primary care in Japan? |
|  |  | This concludes the interview. Thank you for your cooperation. |

Structured Interview Questions **For Clinical Audit + Coaching Group**

| Category | Sub category | Question |
| --- | --- | --- |
| Opening | Ice breaking | Thank you for the participation of the interview. Do you have enough time and space? Please relax... |
|  | Recording + Consent | Obtain consent |
|  | Original experience and thoughts on appropriate usage of BZRAs and QI | Before this intervention begins, tell us your thoughts and experiences regarding the appropriate usage of BZRAs and Quality Improvement in medicine? |
| Clinical Audit + Coaching | Experience(of Audit　and coaching) | Please recall the intervention you received for this study. What intervention did you actually receive? What did you think or feel about the intervention at the time? (Please display the QI Monthly Report and PDSA Summary for each site on the screen.) |
|  | Probe | Audit:  How did you feel about receiving the data?  What did you think of the way the reports were sent?  How much did you actually look at the report?  What did you think when you saw the contents (graphs/reports)?  How easy to read?  What did you think about the appropriateness of the indicators?  Coaching:  What did you think of the intervention methods (frequency and format)?  What did you think of the intervention content (QI concept, methodology, tools, etc.)?  What did you think of the feedback and consultation part of the facility's QI activities? |
|  | Experience(of QI) | What did you actually do as QI activities during the intervention? What did you actually do as QI activities during the intervention? |
|  | Probe | Personally? As a team? Have there been any changes to the system? |
|  | Feasibility | Feasibility (Is it realistically possible to continue Clinical Audit and/or Coaching as part of your daily work in the future? (i.e., can you participate in it without difficulty?) What do you think about the feasibility of Clinical Audit and/or Coaching? |
|  | Probe | How about dividing it into Audit and Coaching? |
|  | Enabler / Effectiveness | What do you think about the benefits and effectiveness? |
|  | Probe | How about dividing it into Audit and Coaching? |
|  | Barrier / Problem | What do you think about barriers? workload? mentality? |
|  | Probe | How about dividing it into Audit and Coaching? |
| Closing | Open question | Is there anything you would like to add at the end? Please feel free to share anything you would like to state based on this experience. |
|  | Probe | What are your thoughts on implementing (systematic and organized) QI activities in primary care in Japan? What do you think is needed to improve the quality in primary care in Japan? |
|  |  | This concludes the interview. Thank you for your cooperation. |
